# Association between Dietary Habits and *Helicobacter pylori* Infection among Bahraini Adults

**DOI:** 10.3390/nu14194215

**Published:** 2022-10-10

**Authors:** Fatema Habbash, Tariq Abdulkarim Alalwan, Simone Perna, Naila Ahmed, Omar Sharif, Adel Al Sayyad, Clara Gasparri, Cinzia Ferraris, Mariangela Rondanelli

**Affiliations:** 1 Department of Family and Community Medicine, Arabian Gulf University, Manama 329, Bahrain; 2 Family and Community Medicine, Internal Medicine Department, King Abdullah Medical City, Manama 328, Bahrain; 3 Department of Biology, College of Science, University of Bahrain, Sakhir P.O. Box 32038, Bahrain; 4 Department of Gastroenterology, King Hamad University Hospital, Muharraq 228, Bahrain; 5 Department of Internal Medicine, The Royal College of Surgeons in Ireland, Muharraq 228, Bahrain; 6 Public Health, Ministry of Health, Manama 323, Bahrain; 7 Endocrinology and Nutrition Unit, Azienda di Servizi alla Persona ‘‘Istituto Santa Margherita’’, University of Pavia, 27100 Pavia, Italy; 8 Laboratory of Food Education and Sport Nutrition, Department of Public Health, Experimental and Forensic Medicine, University of Pavia, 27100 Pavia, Italy; 9 IRCCS Mondino Foundation, 27100 Pavia, Italy; 10 Unit of Human and Clinical Nutrition, Department of Public Health, Experimental and Forensic Medicine, University of Pavia, 27100 Pavia, Italy

**Keywords:** dietary habits, *Helicobacter pylori*, socio-demographic factors, biochemical measurement, vitamin D, Bahrain

## Abstract

Helicobacter pylori (*H. Pylori*) infection is the main bacterial cause of several gastrointestinal disorders. This study aims to estimate the prevalence of *H. pylori* infection in a population of Bahraini adults seeking care in gastroenterology clinics in a tertiary care hospital in the Kingdom of Bahrain and examine the association between dietary habits and other factors with *H. pylori* infection. The study is a hospital-based retrospective, cross-sectional analytical study that included 200 participants. *H. pylori* infection prevalence among the studied group was 55.5%, and it was significantly higher among participants with a high school education or less (44.1%). Among dietary habits, the mean of frequency of green tea, coffee and honey intake was significantly lower among the *H. pylori* infected participants compared to their non-infected counterparts. *H. pylori* infection was significantly higher among participants with vitamin D deficiency (63.6%) compared to participants with normal vitamin D (30%) (*p* = 0.001) and each unit decrease in serum vitamin D was associated with an increased risk of infection by 1.1 times (OR = 1.1; 95% CI: 1.05, 1.18; *p* < 0.001). The study revealed that high educational levels, consumption of honey, green tea, and coffee, as well as normal serum vitamin D level, were independent protectors against *H. pylori* infection. Additional studies are needed to estimate the prevalence and predisposing factors of *H. pylori* infection in the general population.

## 1. Introduction

*Helicobacter pylori* (*H. pylori*) is a Gram-negative spiral-shaped bacterium, which colonizes and grows in human gastric epithelial tissue and mucosa [1]. More than 50% of the global population are infected by *H. pylori* especially in developing countries and among populations with low socioeconomic status [2]. *H. pylori* is usually transmitted through the feco-oral route due to ingestion of contaminated water or food, but it can be transmitted through direct contact with saliva and vomitus [3,4]. The microorganism was classified as a group 1 carcinogen [5] and it causes various upper gastrointestinal (GI) disorders including gastritis, gastroduodenal ulcer diseases, and gastric adenocarcinoma [6]. The latter was recognized to be the fourth-leading cause of cancer-related deaths worldwide in 2020 [7].

Acquisition and various disease outcomes of *H. pylori* infection are intermediated by complex interactions between bacterial virulence, host, and environmental factors [8]. There is a high level of disparity in *H. pylori* genetic recombination and these genetic differences might exist even in *H. pylori* colonizing the same individual [9]. Host genetic background might contribute to protection from infection with *H. pylori* infection [9]. Some factors associated with *H. pylori* infection include age, gender, ethnicity, educational level, and household income [10]. Furthermore, the crowding index, living standards which include sanitation and hygiene, and the source of drinking water have all been shown to be associated with H. *pylori* infection [11]. Findings related to the relationship between smoking and *H. pylori* infection in previous studies are conflicting [12,13,14,15].

Over the past years, epidemiological studies have found that diet plays a significant role in the development of *H. pylori* infection and investigated the association between the intake of certain foods and nutrients and the development of such infection [16,17,18]. Some studies have reported that salty, pickled, fermented, or smoked foods increased the risk of *H. pylori* infection [19,20]. On the other hand, other studies have shown that antioxidant-rich fruits and vegetables were protective against *H. pylori* infection [21,22,23]. Moreover, it was reported that lower intakes of raw vegetables were significantly associated with a higher risk of *H. pylori* infection [17]. Similarly, meat consumption and consumption of restaurant food were associated in some studies with an increased risk of *H. pylori* infection, while chili pepper intake was shown to have a protective effect [12,17]. In addition, some studies revealed a protective effect of honey and green tea consumption against *H. pylori* infection [24,25,26]. Coffee consumption has been linked to several health benefits and some studies found an inverse association between coffee consumption and the systemic levels of some inflammatory markers [27]. However, it was reported that frequent consumption of coffee was associated with an increased rate of *H. pylori* infection and exacerbation of *H. pylori*-related gastritis symptoms [18,28]. Some studies did not find any association between coffee consumption and *H. pylori* infection [10,29,30]. In addition, the relationship between *H. pylori* infection and several modifiable cardio-metabolic risk factors was reported in the literature [31,32,33].

Risk factors associated with *H. pylori* infection, especially lifestyle and dietary habits, have not been investigated thoroughly in the Kingdom of Bahrain. Given the high burden of *H. pylori* infection in developing countries and the high prevalence of modifiable cardio-metabolic risk factors in the Middle East and North Africa (MENA) region, including the Kingdom of Bahrain, a study investigating the relationship between predisposing factors to *H. pylori* infection including dietary habits is warranted. This study aims to provide preliminary data regarding *H. pylori* infection prevalence and predisposing factors among a group of Bahraini adults followed in the Gastroenterology (GE) unit in a tertiary care hospital. The findings of this study will help in the future planning of appropriate preventive, diagnostic, and treatment strategies for *H. pylori* infection.

## 2. Materials and Methods

### 2.1. Study Design, Setting and Duration

This hospital-based retrospective cross-sectional, analytical study was conducted in the Gastroenterology unit in King Hamad University Hospital (KHUH) in the Kingdom of Bahrain between the period of January and September 2021. It combines data from medical records for *H. pylori* status, other comorbidities, and biochemical parameters with sociodemographic, lifestyle, and dietary habits information using a tele-interview.

### 2.2. Study Participants

Participants were recruited if they were 18 years or above, Bahraini, following treatment in GE unit in KHUH, and had done *H. pylori* testing within the previous 18 months with either upper GI tract endoscopy biopsy testing or UBT, or both. Patients with updated medical records within the past 18 months were eligible. Patients were excluded if they had a history and/or documentation of *H. pylori* eradication therapy prior to *H. pylori* testing, had a previous diagnosis of cancer, inflammatory diseases such as coeliac disease, inflammatory bowel disease, or certain food allergies, or had a history of gastric or intestinal surgery or previous gastric perforation or hemorrhage. Patients who were tested for *H. pylori* status by methods other than upper GI tract endoscopy biopsy testing or UBT were not included. Women who were pregnant at the time of the study or previously pregnant within the past 18 months were excluded since their dietary habits might be changed during pregnancy.

### 2.3. Sample Size

Sample size was calculated assuming the following parameters: alpha error = 0.05, power = 80%, expected effect size: odds ratio (OR) = 1.4 (for the diet as a risk factor), prevalence of *H. pylori* (outcome) = 0.50. A total of 200 patients were included in the study.

### 2.4. Research Tools

A validated structured questionnaire was used as the instrument for data collection.

The questionnaire contains four sections to collect data on sociodemographic, lifestyle, dietary habits, and medical conditions and biomarkers. Assessment of dietary habits and the frequency of consumption of food and beverages items were assessed by combining a validated short version FFQ that was used in previous studies [10,34], the Bahraini FFQ which is in process of validation, and some food and beverages included based on findings from the literature (honey, green tea, and soft drinks). The frequency of consumption for the past 18 months was assessed by selecting one of five categories “less than once per month/none”, “1–2 times/month”, “1–2 times/week”, “3–4 times/week” and “every day”. The last section involved data collected from the participant’s medical records which included documentation of comorbidities such as type 2 diabetes (T2D), hypertension and hyperlipidemia, systolic and diastolic blood pressure (BP) (mmHg), height (cm), and weight (kg), FBS (mmol/L), HbA1c as a percentage (DCCT unit), total cholesterol (mmol/L), low-density lipoprotein (LDL) (mmol/L), high-density lipoprotein (HDL) (mmol/L), triglycerides (TG) (mmol/L) and vitamin D level (ng/mL). *H. pylori* status (positive vs. negative) was determined upon the result of either UBT, upper GI tract biopsy testing, or both, which had been done within the previous 18 months. The method used for diagnosis was recorded.

### 2.5. Data Collection and Procedures

A structured electronic questionnaire was used by five trained interviewers during telephone interviews to document the consent of participants and collect data related to sociodemographic, lifestyle, and dietary habits. *H. pylori* status and medical data were retrieved from the medical records and documented in the electronic questionnaire form prior to submission. All questionnaires were collected centrally by the main investigator to ensure confidentiality.

### 2.6. Data Entry

Information obtained on anthropometric and biochemical measurements were recoded as binary (normal level versus not) based on widely known cutoff levels for each parameter. These BMI categorization and cut-point values for biomarkers were based on international and national recommendations [35,36,37,38,39].

### 2.7. Statistical Analysis

All data were entered and analyzed using the Statistical Package for the Social Sciences (SPSS) version 26 (Chicago, IL, USA) software. Categorical variables were computed as frequencies and percentages, and continuous (numerical) variables were computed as mean and standard deviation. Student’s t-test was conducted to examine differences of means. The Chi-Square test was used to compare frequency distributions of categorical variables. The frequency of food and beverages consumption per week was converted into a numerical scale (less than once per month/None = 0, 1–2 times/month = 1, 1–2 times/week = 2, 3–4 times/week = 4, and everyday = 7). The mean for each item was calculated for *H. pylori* positive and negative groups. Univariate logistic regression was employed to evaluate the crude association between dietary factors and *H. pylori* status. Binary logistic regression was used to explore the risk factors that affect the presence of *H. pylori*. The odds ratio (OR) was calculated using a 95% confidence interval (CI). A *p*-value of ≤0.05 was statistically significant in all statistical tests used. The internal consistency reliability using Cronbach’s alpha coefficient was used to verify the reliability of the food frequency questionnaire.

## 3. Results

### 3.1. The Prevalence of H. pylori Infection among the Study Participants

In the present study, 200 participants were recruited. The prevalence of *H. pylori* infection among the study population was 55.5% (95% CI: 48.7%, 62.3%). *H. pylori* status was determined in more than half of the participants (51%) by gastric biopsy testing, 35.5% by a urea breath test, and 13.5% by both methods (Table S1).

### 3.2. Sociodemographic Characteristics of the Study Participants

Table 1 presents the sociodemographic characteristics of the study participants and the association between the sociodemographic characteristics and *H. pylori* infection. The age range of the participants was between 18 to 79 years and the mean age was 51.4 years (95% CI: 49.5–53.3). The proportion of females was larger than males (57% vs. 43%). The majority of participants were married (80%) and with a high school education or less (70.5%).

### 3.3. Association of Sociodemographic Characteristics and Lifestyle Factors with H. pylori Infection

*H. pylori* infection was significantly higher among participants with high school education or less compared to those with college/university education (*p* = 0. 035) (Table 1). *H. pylori* infection was more prevalent among smokers (73.9%) compared to non-smokers (53.1%); however, the difference was not statistically significant (Table 2). There was no significant association between *H. pylori* infection and any of the lifestyle factors investigated in this study (Table 2). 

### 3.4. Association between Dietary Habits and H. pylori Infection

Table 3 demonstrates the differences and associations between the frequency of consumption of food and beverages in *H. pylori* positive and negative participants. There was a significant negative relationship between the mean level of green tea (*p* = 0.012), honey (*p* = 0.018), and coffee consumption (*p* = 0.007) with *H. pylori* infection. The mean of frequency of green tea, coffee, and honey intake was significantly lower among the *H. pylori* infected participants compared to their non-infected counterparts. There was no significant association between the mean level of frequency of consumption of other food and beverage items and *H. pylori* infection.

Table 4 shows that the *H. pylori* positivity rate was significantly lower (38.6%) in green tea consumers ≥ 1 day/week compared with their counterparts (60.3%) (*p* = 0.0011). Logistic regression analysis showed a lower risk of *H. pylori* infection in participants who consume green tea ≥ 1 day/week (OR, 0.011; 95% CI, 0.23–0.92). *H. pylori* positivity rate was lower (50%) in coffee consumers ≥ 1 day/week compared with their counterparts (63.4%) and in honey consumers ≥ 1 day/week (48.8%) compared with the other participants (60.5%), but this difference was not statistically significant (Table 4).

As shown in Table 5, *H. pylori* infection was more prevalent among participants who consumed well water during childhood as the main source of drinking water (66%), chili peppers (58.8%), salty foods (60.6%), and restaurant meals more than three times a week (59%); however, the differences were not statistically significant.

### 3.5. Association of Some Medical Conditions and Biomarkers of the Study Participants with H. pylori Infection

Table 6 shows some of the medical conditions and biochemical markers of the study participants and their association with *H. pylori* infection. *H. pylori* positivity rate among participants who were overweight (58.9%) or obese (50.50.5%) was higher compared to participants with normal BMI (48.1%); however, this difference was not statistically significant. The proportion of *H. pylori* was higher among participants with hyperlipidemia (61.7%), and abnormally high levels of LDL (57.7%) as compared to their counterparts. *H. pylori* infection was significantly more prevalent among participants with vitamin D deficiency (63.6%) compared to participants with normal vitamin D levels (30%) (*p* = 0.001).

### 3.6. Bivariate Logistic Regression Analysis of Some of the Variables 

Table 7 gives an overview of the results of the bivariate logistic regression analysis. Participants with a high school degree or below were 1.38 times more likely to develop *H. pylori* infection compared to participants with a college/university degree, but the difference is not statistically significant (*p* = 0.474). In addition, smokers were 3.58 times more likely to develop *H. pylori* compared to non-smoker participants, but the difference is not statistically significant (*p* = 0.129). Finally, the risk of *H. pylori* infection increases by 1.11 per one unit decrease of vitamin D (OR: 1.11, 95% CI of 1.05, 1.18, *p* < 0.001).

## 4. Discussion

The present study estimated the prevalence of *H. pylori* infection in a population of Bahraini adults seeking care at tertiary level and investigated the relationship between several factors including dietary habits and *H. pylori* infection. The overall prevalence of *H. pylori* infection found in this study was 55.5%. This is comparable to the prevalence of 59.4% reported by Alshaikh et al. (2021) in a recent retrospective study conducted in the Kingdom of Bahrain [40]. Interestingly, previous studies conducted more than 20 years back on samples of dyspeptic adult patients who underwent gastroscopy in a tertiary care hospital in Bahrain revealed prevalence ranges between 75% and 79.4% [41,42]. These findings could suggest a decreasing trend of *H. pylori* infection among symptomatic patients specifically, which raises a question if that the decreasing trend of *H. pylori* infection observed in symptomatic patients applies to the general population of Bahrain. The prevalence found in this study is near to the prevalence of 52.4% reported by Assaad et al. (2018) in a study conducted in Lebanese patients referred for upper GI endoscopy [10]. However, a lower prevalence was reported in studies conducted in dyspeptic patients in Oman (41%) and in Jazan Province in Saudi Arabia (46.5%) [30,43]. Many studies conducted in the Middle East/North Africa (MENA) region including Iran, Egypt, and Turkey reported higher infection rates which reached 86.8% in Iran [14,31,44]. The prevalence of *H. pylori* among subjects with dyspepsia in the United States, Brazil, and China was 28.9%, 57%, and 84% respectively [43]. The difference in the prevalence of *H. pylori* infection observed in different studies might be due to variation in the study design, sample size, study setting, the period in which the study was conducted, participants’ characteristics, ethnicities of the sample, and testing methods used to determine *H. pylori* status. In addition, variation in bacterial virulence and stereotypes, antibiotic resistance, environment, living standards, socioeconomic and lifestyle factors, and dietary habits in different contexts could affect this prevalence. The prevalence reported in this study cannot be generalized to reflect *H. pylori* prevalence in the Kingdom of Bahrain, since it represents only the rate of the infection in a relatively small cohort of patients who were following treatment in one of the tertiary hospitals in the country.

*H. pylori* infection was significantly higher among participants with lower educational levels (high school degree or below) (60.3% positivity rate) compared to subjects with higher educational levels (college/university degrees) (44.1% positivity rate). Participants with university degrees might be more knowledgeable/aware of health-related issues and have a healthier lifestyle compared to those with lesser educational levels. In agreement with this study’s finding, participants with higher educational levels were less likely to have *H. pylori* infection in studies conducted in Turkey, Korea, and China [14,45]. 

None of the lifestyle factors studied was associated with *H. pylori* infection. This finding could be due to the small sample size, participants’ characteristics as patients with certain health-seeking behavior, and the study design which is prone to recall bias. Furthermore, the data was collected during the COVID-19 pandemic in which the lifestyle of the majority of the population has been changed due to the quarantine and social distancing precautions [45]. Consistent with this finding, Assaad et al. (2018) in Lebanon found no association between *H. pylori* infection and any of the lifestyle factors studied which include smoking, alcohol consumption, physical activity, number of sleep hours per night, and perceived level of stress [10].

Findings in this study revealed that the *H. pylori* infection rate was lesser among participants with higher consumption of green tea and honey. *H. pylori* infection rate was significantly lesser among participants who consume green tea one time or more per week. Green tea and honey have been shown to exhibit antibacterial activity to inhibit the growth of *H. pylori* and gastric mucosal inflammation [46,47]. Honey has a potent antibacterial activity due to certain characteristics as low pH, high osmolarity and hydrogen peroxide content [47,48]. Consistent with this finding, a study conducted in Bulgaria to assess the dietary habits of 150 patients with dyspepsia revealed that honey intake at least once a week (OR: 0.38) and green/black tea consumption for at least one day or more a week (OR: 0.45) were significantly associated with lower prevalence of *H. pylori* infection [24]. Similarly, Mard et al. (2014) and Yordanov et al. (2017) found a significant negative correlation between the intake of honey and *H. pylori* infection [25,26]. This study also showed that *H. pylori* infection was associated with lower frequencies of coffee consumption. Coffee consumption has been linked to several health benefits as lowering the risk of some diseases such as cardiovascular diseases, type 2 diabetes, obesity, and some types of cancers [49]. Coffee is rich in polyphenols which are known to affect immune function and chronic inflammation [27]. It also contains arabinogalactan proteins which are a type of polysaccharide that exhibits prebiotic and immunomodulatory properties [49]. Loftfield et al. (2015) found an inverse association between coffee consumption and the systemic levels of some inflammatory markers [27]. Findings from the literature on the relationship between coffee consumption and *H. pylori* infection are inconsistent. Alebie et al. (2016) in a study that included 145 Ethiopian students with gastritis found that consumption of coffee exacerbates *H. pylori* related gastritis symptoms [28]. Monno et al. (2019) in a retrospective study conducted in Italy revealed that the frequent consumption of coffee increases the *H. pylori* infection rate [18], while other studies did not find any association between coffee consumption and *H. pylori* infection [10,29,30]. The relationship between coffee consumption and *H. pylori* infection might be attributed to the differences in the type of coffee consumed and preparation methods. Another reason for the observed finding could be an intentional reduction of coffee consumption among *H. pylori* infected participants due to their personal beliefs or health care workers’ instructions. It might be important in future studies to include data about the type and amount of coffee consumed, preparation methods and if the participant intentionally altered consumption for any reason.

It is essential to study details about dietary patterns, since evaluating food items in isolation might not provide a full view of nutrients’ interaction. Moreover, dietary habits were assessed during the COVID-19 pandemic, during which some dietary habits might have been altered [50]. The COVID-19 pandemic affected eating behaviors and limited access to fresh food due to quarantine precautions and lockdown which led to increased consumption of processed and fast foods that are rich in salt, sugar, and saturated fat [51].

This study showed that *H. pylori* infection was more prevalent among participants with vitamin D deficiency. Evidence from the recent literature indicates that vitamin D possesses immunoregulatory functions that exhibit an effect on susceptibility to infections in general and to *H. pylori* specifically [52]. Vitamin D might decrease the risk of infection by various mechanisms; vitamin D improves innate immunity by modulating the production of antimicrobial peptides and cytokine response [52]. Furthermore, Vitamin D helps to enhance the activity of monocytes and macrophages and contributes to systemic antimicrobial effects [53]. Consistent with this study’s findings, a multi-centric study reported that *H. pylori* infected participants had significantly lower serum vitamin D levels compared to the non-infected group [54]. Assaad et al. (2018) in Lebanon, reported that *H. pylori* infection risk was significantly higher among participants with vitamin D deficiency (OR = 29.14) compared to participants with normal vitamin D levels [10]. A recent study conducted in Turkey revealed that vitamin D deficiency was associated with increased odds of *H. pylori* infection by almost 3 times [55]. Yang et al. (2019) revealed that vitamin D had a protective effect against *H. pylori* infection and improved the success rate of *H. pylori* eradication [52]. The relationship between vitamin D and *H. pylori* infection is worth more investigation in the context of Bahrain, as many factors might be involved including diet and comorbidities. Considering vitamin D supplementation as part of prevention and treatment plans of *H. pylori* infection for certain groups in the population might be effective.

## 5. Strengths and Limitations

This study had several strengths and limitations. First, to the best of our knowledge, this is the first study in the Kingdom of Bahrain and one of few in the region to evaluate the association between sociodemographics, lifestyle, dietary habits, and some medical conditions with *H. pylori* infection. Second, a short version-13 item-FFQ previously validated by Yassibas [31] was used to assess dietary intake, FFQ is considered one of the best dietary tools to assess the relationship between diet and disease. Furthermore, the internal consistency and reliability of this tool were improved by adding items from the Bahraini FFQ that is in process of validation and other food items and beverages that were related to *H. pylori* infection in previous studies. Third, the data were collected through telephone interviews and not self-administered, so that the interviewer might clarify any misunderstandings if needed and minimize missing information. Fourth, *H. pylori* status was determined upon upper GI endoscopy biopsy testing and/or UBT, both of which have high diagnostic accuracy. Finally, medical data were retrieved from the patients’ medical records, minimizing any self-reporting or categorization bias. Some limitations regarding this study should be considered when interpreting the results. The data collection was conducted during the COVID-19 pandemic; within this period some lifestyle and dietary habits might be affected. In addition, due to the regulations related to that period, some non-urgent investigations/procedures were rescheduled, which affected our reach to the targeted population. Due to that reason, we included any patient who had done the *H. pylori* testing within the previous 18 months by either UBT or upper GI endoscopy biopsy testing. The convenience sampling method used to select the participants and this subgroup characteristics might limit the ability to generalize the results to the general population. Moreover, the use of FFQ might represent some limitations. Food intake in the previous 18 months of the interview was self-reported by participants with no measure for verification, which might lead to possible recall and information bias. In addition, intake of food and beverage items was assessed without specifying quantities or portion size. However, the variation in portion size between participants is smaller than the variation in the frequency of consumption, which will have a limited effect on the findings. Some medical data were missing or not updated for a group of participants. This could contribute to the final findings. Finally, an inference of causality cannot be generated due to the cross-sectional study design.

## 6. Conclusions

*H. pylori* infection is a major public health problem that affects more than half of the world population leading to a range of GI and extra-gastric problems. This study is the first in Bahrain and one of few in the region to investigate the relationship between diet and *H. pylori* infection. *H. pylori* infection was significantly higher among participants with lower educational levels (high school degree or below) compared to those with higher educational levels (university degree). Intake of honey, green tea, and coffee was found to be protective against *H. pylori* infection. In addition, vitamin D deficiency was a risk factor for *H. pylori* infection. Including diet, in prevention measures and in support of treatment options of *H. pylori* infection will provide an acceptable convenient approach to control *H. pylori* with reasonable cost, high availability, and lesser side effects compared to medications.

## Figures and Tables

**Table 1:** Sociodemographic characteristics and their association with *H. pylori* status among the study participants.

Sociodemographic Characteristics		*H. pylori* Status		Chi-Square *p*-Value
	*n* (%)	Positive	Negative	
		*n* (%)	*n* (%)	
Age				
18–30 years	17 (8.5)	10 (58.8)	7 (41.2)	0.980
31–45 years	42 (21.0)	23 (54.8)	19 (45.2)	
46–60 years	83 (41.5)	45 (54.2)	38 (45.8)	
Older than 60 years	58 (29.0)	33 (56.9)	25 (43.1)	
Gender				
Male	86 (43.0)	48 (55.8)	38 (44.2)	0.938
Female	114 (57.0)	63 (55.3)	51 (44.7)	
Marital status				
Married	160 (80.0)	88 (55)	72 (45)	0.776
Unmarried	40 (20.0)	23 (57.5)	17 (42.5)	
Educational level				
High school or below	141 (70.5)	85 (60.3)	56 (39.7)	**0.035**
College/university	59 (29.5)	26 (44.1)	33 (55.9)	
Employment status				
Employed	49 (24.5)	24 (49)	25 (51)	0.277
Unemployed	81(40.5)	43 (53.1)	38 (46.9)	
Retired	70 (35.0)	44 (62.9)	26 (37.1)	
Household income				
BHD 300 or less	17(8.5)	16 (59.3)	11 (40.7)	0.201
BHD 301–600	61 (30.5)	35 (57.4)	26 (42.6)	
BHD 601–900	59 (29.5)	37 (62.7)	22 (37.3)	
BHD 900 or more	53 (26.5)	23 (43.4)	30 (56.6)	
Number of household members				
1–4	79 (39.5)	44 (55.7)	35 (44.3)	0.493
5–8	90 (45.0)	47 (52.2)	43 (47.8)	
8 or greater	31 (15.5)	20 (64.5)	11 (35.5)	
Number of household rooms				
1–3	69 (34.5)	37 (53.6)	32 (46.4)	0.858
4–6	96 (48.0)	54 (56.3)	42 (43.8)	
6 or more	32 (16.0)	19 (59.4)	13 (40.6)	

Data in bold are statistically significant.

**Table 2:** Lifestyle factors and their association with *H. pylori* status among the study participants.

		*H. pylori* Status		Chi-Square *p*-Value
Lifestyle Factors	*n* (%)	Positive	Negative	
		*n* (%)	*n* (%)	
Smoking status				
Non-smoker	177 (88.5)	94 (53.1)	83 (46.9)	0.059
Smoker	23 (11.5)	17 (73.9)	6 (26.1)	
Number of cigarettes smoked/day				
10 or less	17 (36.9)	8 (47.1)	9 (52.9)	0.560
11–20	17 (37.0)	10 (58.8)	7 (41.2)	
20 or more	12 (26.1)	8 (66.7)	4 (33.3)	
Level of perceived stress				
Less than 3 (Low)	30 (15)	18 (60)	12 (40)	0.861
3–6 (Moderate)	89 (44.5)	49 (55.1)	40 (44.9)	
7–10 (High)	81 (40.5)	44 (54.3)	37 (45.7)	
Number of hours of sleep per night				
Less than 5 h	39 (19.5)	25 (64.1)	14 (35.9)	0.186
5–7 h	123 (61.5)	62 (50.4)	61 (49.6)	
More than 7 h	38 (19)	24 (63.2)	14 (36.8)	
Number of times/week of being physically active				
None	75 (37.5)	42 (56)	33 (44)	0.427
Less than 1 time per week	22 (11.0)	12 (54.5)	10 (45.5)	
1–3 times per week	21 (10.5)	15 (71.4)	6 (28.6)	
More than 3 times per week	82 (41.0)	42 (51.2)	40 (48.8)	
Duration of physical activity				
Less than 20 min	21 (16.8)	11 (52.4)	10 (47.6)	0.776
20 min or more	104 (83.2)	58 (55.8)	46 (44.2)	

**Table 3:** Mean level of frequency of consumption of food and beverage items and their association with *H. pylori* status.

Food	*H. pylori* Status		Mann–Whitney *p*-Value
	Positive	Negative	
	Mean ± SD	Mean ± SD	
Grains	6.3 ± 1.6	6.0 ± 1.9	0.374
Green vegetables	4.7 ± 2.6	5.4 ± 2.3	0.057
Tuberous vegetables	4.4 ± 2.5	4.9 ± 2.3	0.153
Fish	2.2 ± 1.4	2.2 ± 1.2	0.746
Chicken	3.9 ± 2.1	4.3 ± 2.0	0.166
Red meat	1.6 ± 1.2	1.8 ± 1.1	0.139
Sausage	0.3 ± 0.8	0.2 ± 0.5	0.740
Hot dog	0.1 ± 0.5	0.2 ± 0.5	0.220
Salami or ham	0.1 ± 0.5	0.3 ± 0.9	0.134
Hamburger	0.8 ± 1.1	0.8 ± 1.2	0.922
Milk	3.3 ± 2.9	3.8 ± 3.0	0.263
Yogurt	3.4 ± 2.7	3.6 ± 2.6	0.374
Salty cheese	3.6 ± 2.7	4.1 ± 2.7	0.182
Fresh fruits	4.9 ± 2.5	5.1 ± 2.4	0.651
Legumes	1.8 ± 1.5	1.7 ± 1.8	0.215
Eggs	3.1 ± 2.3	3.2 ± 2.2	0.571
Nuts and dried fruits	2.5 ± 2.3	3.2 ± 2.7	0.154
Salted fish	0.3 ± 0.7	0.6 ± 1.1	0.130
Pickled vegetables	1.0 ± 1.9	0.7 ± 1.3	0.508
Onion	4.4 ± 2.8	4.4 ± 2.8	1.000
Garlic	4.0 ± 2.9	4.0 ± 3.0	0.926
Tomato	4.8 ± 2.6	4.7 ± 2.8	0.828
Butter and ghee	1.4 ± 2.0	1.6 ± 2.2	0.377
Vegetable oils	5.4 ± 2.4	5.6 ± 2.3	0.561
Deserts	2.7 ± 2.6	2.7 ± 2.5	0.835
Tea	4.6 ± 3.0	5.1 ± 2.7	0.214
Green tea	0.8 ± 1.8	1.3 ± 2.1	**0.012**
Coffee	2.7 ± 2.9	3.7 ± 3.0	**0.007**
Soft drinks	1.4 ± 2.2	1.1 ± 1.8	0.350
Honey	1.8 ± 2.3	2.8 ± 2.9	**0.018**

Data in bold are statistically significant.

**Table 4:** Association between *H. pylori* status with green tea, coffee, and honey consumption.

	*H. pylori* Status		Chi-Square *p*-Value	OR	*p*-Value	95% CI for OR
	Positive	Negative				
	*n* (%)	*n* (%)				
Green tea						
<1 day weekly	94 (60.3)	62 (39.7)	**0.011**	0.46	**0.029**	(0.23, 0.92)
≥1 day weekly	17 (38.6)	27 (61.4)				
Coffee						
<1 day weekly	52 (63.4)	30 (36.6)	0.060	0.62	0.107	(0.34, 1.11)
≥1 day weekly	59 (50)	59 (50)				
Honey						
<1 day weekly	69 (60.5)	45 (39.5)	0.100	0.65	0.149	(0.37, 1.17)
≥1 day weekly	42 (48.8)	44 (51.2)				

Data in bold are statistically significant.

**Table 5:** Dietary habits of the study participants and their association with *H. pylori* status.

Dietary Factors	N (%)	*H. pylori* Status		Chi-Square *p*-Value
		Positive	Negative	
		*n* (%)	*n* (%)	
Source of drinking water during childhood				
Tap water	115 (57.5)	62 (53.9)	53 (46.1)	0.117
Well water	53 (26.5)	35 (66)	18 (34)	
Filtered or mineral water	32 (16.0)	14 (43.8)	18 (56.3)	
Consumption of chili pepper				
Yes	102 (51.0)	60 (58.8)	42 (41.2)	0.335
No	98 (49.0)	51 (52)	47 (48)	
Salt status of consumed dishes				
Salty	99 (49.5)	60 (60.6)	39 (39.4)	0.150
Less salty/Salt free	101 (50.5)	51 (50.5)	50 (49.5)	
Speed of meals consumption				
Fast	61 (30.5)	33 (54.1)	28 (45.9)	0.883
Normal	78 (39.0)	45 (57.7)	33 (42.3)	
Slow	61 (30.5)	33 (54.1)	28 (45.9)	
Temperature status of meals consumed				
Cool/warm	83 (41.5)	45 (54.2)	38 (45.8)	0.758
Hot	117 (58.5)	66 (56.4)	51 (43.6)	
Frequency of consuming meals prepared outside home				
<1 time per month/Never	54 (27.0)	30 (55.6)	24 (44.4)	0.897
1–3 times/month	60 (30.0)	34 (56.7)	26 (43.3)	
1–2 times per week	47 (23.5)	24 (51.1)	23 (48.9)	
3–7 times per week	39 (19.5)	23 (59)	16 (41)	

**Table 6:** Medical condition of the study participants and its association with *H. pylori* status.

Medical Characteristics	N (%)	*H. pylori* Status		Chi-Square *p*-Value
		Positive	Negative	
		*n* (%)	*n* (%)	
Diagnosis of Hypertension				
Yes	67 (33.5)	38 (56.7)	29 (43.3)	0.806
No	133 (66.5)	73 (54.9)	60 (45.1)	
Diagnosis of Diabetes				
Yes	62 (31.0)	35 (56.5)	27 (43.5)	0.856
No	138 (69.0)	76 (55.1)	62 (44.9)	
Diagnosis of Hyperlipidemia				
Yes	60 (30.0)	37 (61.7)	23 (38.3)	0.251
No	140 (70.0)	74 (52.9)	66 (47.1)	
BMI				
Normal weight (18.5–24.9)	27 (15.3)	13 (48.1)	14 (51.9)	0.530
Overweight (25.0–29.9)	56 (31.6)	33 (58.9)	23 (41.1)	
Obese (≥30.0)	93 (52.5)	47 (50.5)	46 (49.5)	
Blood pressure				
Normal	46 (23.2)	25 (54.3)	66 (45.5)	0.851
Above normal	152 (76.8)	85 (55.9)	67 (44.1)	
Cholesterol				
Normal (<5.6 mmol/L)	67 (68.4)	35 (52.2)	32 (47.8)	0.402
Above normal (≥5.6 mmol/L)	31 (31.6)	19 (61.3)	12 (38.7)	
TG				
Normal (<1.7 mmol/L)	70 (72.2)	38 (54.3)	32 (45.7)	0.829
Above normal (≥1.7 mmol/L)	27 (27.8)	14 (51.9)	13 (48.1)	
LDL				
Normal (<2.6 mmol/L)	45 (46.4)	22 (48.9)	23 (51.1)	0.386
Above normal (≥2.6 mmol/L)	52 (53.6)	30 (57.7)	22 (42.3)	
HDL				
Normal (>1.5 mmol/L)	26 (26.8)	17 (65.4)	9 (34.6)	0.159
Below normal (≤1.5 mmol/L)	71 (73.2)	35 (49.3)	36 (50.7)	
FBS				
Normal (<5.6 mmol/L)	18 (45.0)	10 (55.6)	8 (44.4)	0.525
Above normal (≥5.6 mmol/L)	22 (55.0)	10 (45.5)	12 (54.5)	
HbA1c				
Normal (<5.7%)	23 (24.2)	12 (52.2)	11 (47.8)	0.688
Above normal (≥5.7%)	72 (75.8)	41 (56.9)	31 (43.1)	
Vitamin D				
Normal (≥20 nmol/L)	40 (34.2)	12 (30)	28 (70)	**0.001**
Below normal (<20 nmol/L)	77 (65.8)	49 (63.6)	28(36.4)	

BMI: Body Mass Index, TG: Triglycerides, HDL: High-Density Lipoprotein, LDL: Low-Density Lipoprotein, FBS: Fasting Blood Sugar, HbA1: Glycated hemoglobin. Analysis was done in accordance with the available data. BMI = 177, Blood pressure = 198, Cholesterol = 98, Triglycerides = 97, LDL = 97, HDL = 97, FBS = 40, HbA1c = 95, and vitamin D = 117. The data in bold is statistically significant.

**Table 7:** Binary logistic regression of educational level, smoking status, and Vitamin D level on *H. pylori* status.

	Odd Ratio	*p*-Value	95% CI for Odd Ratio
Educational level			
High school or below	1.38	0.474	(0.57, 3.35)
College/university	Reference		
Smoking status			
Smoker	3.58	0.129	(0.69, 18.51)
Non-smoker	Reference		
Vitamin D	1.11	**<0.001**	(1.05, 1.18)

Data in bold is statistically significant.

## Data Availability

Data supporting reported results is not publicly archived, if required, it can be provided by the principal investigator.

## Supplementary Materials

The following are available online at https://www.mdpi.com/article/10.3390/nu14194215/s1, Table S1: Frequencies and distribution of H. pylori diagnostic methods used; Table S2: Percent distribution of frequency of consumption of food and beverage items by the study participants.
