# Supplementary material for: Association between Dietary Habits and Helicobacter pylori Infection among Bahraini Adults
Source: Nutrients. 2022 Oct 10;14(19):4215. doi: 10.3390/nu14194215 (PMC9572631; doi:10.3390/nu14194215)
Supplement: Supplementary file 1 [file nutrients-14-04215-s001.zip › nutrients-1938487-supplementary.pdf]

Table S1. Frequencies and distribution of *H. pylori* diagnostic methods used.

Parameter	*n*	%
**Diagnostic method used**		
Urea breath test	71	35.5
Gastric biopsy testing	102	51.0
Both	27	13.5

Table S2. Percent distribution of frequency of consumption of food and beverage items by the study participants.

Food	<1 time per month/None *n* (%)	1–2 times/month *n* (%)	1–2 times/week *n* (%)	3–4 times/week *n* (%)	Every Day *n* (%)
Grains	4 (2.0)	1 (0.5)	12 (6.0)	23 (11.5)	160 (80.0)
Green vegetable	9 (4.5)	14 (7.0)	33 (16.5)	30 (15.0)	114 (57.0)
Tuberous vegetables	6 (3.0)	18 (9.0)	41 (20.5)	43 (21.5)	92 (46.0)
Fish	16 (8.0)	30 (15.0)	111 (55.5)	39 (19.5)	4 (2.0)
Chicken	8 (4.0)	6 (3.0)	49 (24.5)	85 (42.5)	52 (26.0)
Red meat	34 (17.0)	45 (22.5)	100 (50.0)	19 (9.5)	2 (1.0)
Sausage	171 (85.5)	18 (9.0)	7 (3.5)	4 (2.0)	0 (0.0)
Hot dog	179 (89.5)	16 (8.0)	4 (2.0)	1 (0.5)	0 (0.0)
Salami or ham	178 (89.0)	15 (7.5)	4 (2.0)	2 (1.0)	1 (0.5)
Hamburger	101 (50.5)	56 (28.0)	35 (17.5)	6 (3.0)	2 (1.0)
Milk	50 (25.0)	22 (11.0)	27 (13.5)	26 (13.0)	75 (37.5)
Yogurt	30 (15.0)	24 (12.0)	48 (24.0)	37 (18.5)	61 (30.5)
Salty Cheese	29 (14.5)	18 (9.0)	42 (21.0)	37 (18.5)	74 (37.0)
Fresh Fruits	6 (3.0)	15 (7.5)	36 (18.0)	30 (15.0)	113 (56.5)
Legumes	43 (21.5)	49 (24.5)	76 (38.0)	23 (11.5)	9 (4.5)
Eggs	18 (9.0)	19 (9.5)	82 (41.0)	40 (20.0)	41 (20.5)
Nuts and dried fruits	41 (20.5)	35 (17.5)	51 (25.5)	31 (15.5)	42 (21.0)
Salted fish	151 (75.5)	29 (14.5)	15 (7.5)	4 (2.0)	1 (0.5)
Pickled vegetables	127 (63.5)	28 (14.0)	31 (15.5)	4 (2.0)	10 (5.0)
Onion	28 (14.0)	11 (5.5)	36 (18.0)	29 (14.5)	96 (48.0)
Garlic	43 (21.5)	12 (6.0)	28 (14.0)	30 (15.0)	87 (43.5)
Tomato	20 (10.0)	13 (6.5)	28 (14.0)	30 (15.0)	109 (54.5)
Butter and ghee	98 (49.0)	30 (15.0)	38 (19.0)	17 (8.5)	17 (8.5)
Vegetable oils	7 (3.5)	13 (6.5)	21 (10.5)	23 (11.5)	136 (68.0)
Deserts	40 (20.0)	36 (18.0)	67 (33.5)	12 (6.0)	45 (22.5)
Tea	28 (14.0)	14 (7.0)	24 (12.0)	14 (7.0)	120 (60.0)
Green tea	135 (67.5)	21 (10.5)	22 (11.0)	7 (3.5)	15 (7.5)
Coffee	60 (30.0)	22 (11.0)	37 (18.5)	13 (6.5)	68 (34.0)
Soft drinks	117 (58.5)	24 (12.0)	30 (15.0)	12 (6.0)	17 (8.5)
Honey	76 (38.0)	38 (19.0)	28 (14.0)	20 (10.0)	38 (19.0)
